# Individual differences in environmental sensitivity: associations between cognitive emotion regulation and mental health

**DOI:** 10.3389/fpsyg.2024.1322544

**Published:** 2024-03-08

**Authors:** Kosuke Yano, Kazuo Oishi

**Affiliations:** ^1^Research Center for Youth Education, National Institution For Youth Education, Tokyo, Japan; ^2^Rikkyo University, Tokyo, Japan

**Keywords:** environmental sensitivity, sensory processing sensitivity, mental health, emotion regulation, longitudinal study

## Abstract

**Introduction:**

Environmental sensitivity is defined as the ability to perceive and process internal and external information. Previous studies have suggested that mental health-related factors differ by sensitivity level. This study aimed to investigate whether environmental sensitivity moderates the associations between cognitive emotion regulation strategies (e.g., *rumination* and *blaming others*) and mental health.

**Materials and methods:**

In this three-wave longitudinal study, participants (*N* = 1,233, 585, and 349 at Times 1, 2, and 3, respectively) completed the Cognitive Emotion Regulation Questionnaire-short and Kessler 10 scale at all the measurement points as well as the 10-item version of the Highly Sensitive Person scale and some covariates only at Time 1.

**Results:**

Latent growth model analyses indicated that the *blaming others* strategy had contrastive effects on changes in mental health by sensitivity level; the increase in *refocusing on planning* was associated with improved mental health over time only for highly sensitive individuals; and the *rumination* and *catastrophizing* strategies were the most important risk factors for mental health problems, although their effects differed slightly by sensitivity level.

**Conclusion:**

The associations between some of the cognitive strategies and mental health differ by environmental sensitivity level. Future investigations based on individual differences in sensitivity could provide innovative insights into practices.

## 1 Introduction

In daily life, people often experience negative emotions such as anxiety and anger. These emotions must be dealt with appropriately to enhance well-being and/or promote adaptive behaviors (Gross, 1998). The framework of cognitive emotion regulation comprehensively describes the cognitive strategies that individuals use in response to threatening or stressful life events (Garnefski et al., 2001). Garnefski et al. (2001) assumed that emotion regulation through cognition is inextricably associated with human life and reviewed the literature regarding the cognitive aspects of coping. Consequently, nine conceptually different strategies were extracted: (1) *positive reappraisal*— thinking about attaching a positive meaning to the event in terms of personal growth; (2) *putting into perspective*—comparing an event other events and downgrading the event’s importance; (3) *rumination*—thinking about the feelings and thoughts associated with negative events; (4) *acceptance*—resigning oneself to what has happened; (5) *self-blame*—blaming oneself after experiencing a stressful event; (6) *positive refocusing*—thinking about positive experiences instead of thinking about the actual event; (7) *blaming others*—blaming the environment or another person for a negative event one has experienced; (8) *catastrophizing*—overemphasizing the terror of what one has experienced; and (9) *refocusing on planning*—thinking about what steps to take and how to handle negative events (Garnefski et al., 2001; Garnefski and Kraaij, 2006a). These strategies could be useful targets for intervention (Garnefski et al., 2005). For example, Nelis et al. (2011) suggested that a cognitive behavioral approach could enhance the appropriate use of these strategies.

To measure the nine strategies, the Cognitive Emotion Regulation Questionnaire (CERQ) and its short version (CERQ-short) were developed (Garnefski et al., 2001; Garnefski and Kraaij, 2006a). The original assumption was that five strategies—*positive reappraisal*, *putting into perspective*, *acceptance*, *positive refocusing*, and *refocusing on planning*—were adaptive, whereas *rumination*, *self-blame*, *blaming others*, and *catastrophizing* were maladaptive. However, some empirical studies on the relationship between cognitive strategies and mental health indices (e.g., depression and anxiety) have failed to provide evidence consistent with their theoretical background (Garnefski et al., 2001; Garnefski and Kraaij, 2006b; Potthoff et al., 2016). A possible explanation for these inconsistencies between theory and evidence is that personal and contextual factors moderate the relationship between strategies and mental health (Aldao et al., 2010). Although studies have considered specific populations like foot-and-mouth crises (Garnefski et al., 2005), situations like the COVID-19 lockdown (Rodas et al., 2022), and personal factors such as nationality (Potthoff et al., 2016) and age (Garnefski and Kraaij, 2006b), little attention has been paid to psychological characteristics. However, given that different factors predict depressive tendencies based on personality (Yano et al., 2021b), investigating its moderating role in the associations between cognitive emotion regulation strategies and mental health could provide useful findings for more effective interventions.

Recently, psychologists have suggested that the concept of environmental sensitivity provides key information to mental health researchers and practitioners (Greven et al., 2019; Yano et al., 2021b). Environmental sensitivity is an overarching meta-framework of several psychological theories and concepts, including the differential susceptibility hypothesis (Belsky, 1997), biological sensitivity to context (Boyce and Ellis, 2005), and sensory processing sensitivity (SPS; Aron and Aron, 1997), and is defined as the ability to perceive and process internal and external information (Pluess, 2015; Greven et al., 2019).^1^ The level of sensitivity can be assessed using genetic (e.g., Keers et al., 2016), biological/physiological (e.g., Pluess et al., 2022), and psychological factors (e.g., Slagt et al., 2018). A growing number of studies have focused on SPS, a psychological marker of environmental sensitivity (Greven et al., 2019). SPS is a normally distributed and highly heritable trait (Pluess et al., 2018; Assary et al., 2021), whose core characteristics are the deeper processing of environmental information, showing stronger emotional and physiological reactivity to positive and negative stimuli, having greater awareness of subtle cues, and getting overstimulated more easily (Aron et al., 2012; Homberg et al., 2016). These features could at least partly be captured by two self-reported scales: the Highly Sensitive Person (HSP) scale (Aron and Aron, 1997) and Highly Sensitive Child scale (Pluess et al., 2018). Numerous studies have used these scales and revealed the differences between SPS and other personality constructs (e.g., Lionetti et al., 2019; Iimura et al., 2023).^2^

In view of susceptibility to environmental influences (Pluess, 2015), people scoring high on SPS benefit more from psychological education programs focusing on emotion regulation (Pluess and Boniwell, 2015; Kibe et al., 2020). These findings suggest that the factors strongly associated with mental health differ by SPS level. A previous systematic review found that dysfunctional thoughts such as *rumination* and *catastrophizing* are more important predictors of depression and/or anxiety, particularly for higher-sensitivity individuals (Bratholm Wyller et al., 2017). Lionetti et al.’s (2022) empirical investigation supported this assumption, while emphasizing the moderating role of the parenting environment. Additionally, the results of an open-ended survey conducted to explore the characteristics and effectiveness of coping strategies among university students with high and low sensitivity (Yano et al., 2021a) indicated that although strategies related to emotion regulation were extracted in all the sensitivity groups, their associations with mental health partly differed between the groups. Specifically, while positive thinking (similar to *positive reappraisal*) may be more effective for individuals with higher sensitivity, it may be important for lower-sensitivity individuals to receive emotional and instrumental support from friends who understand their feelings and offer useful advice. Thus, environmental sensitivity could moderate the association between cognitive emotion regulation strategies and mental health, although its moderating effect has been insufficiently examined. Additionally, there are limitations to adapting the existing evidence to practice because of the data being cross-sectional or qualitative (Yano and Oishi, 2018; Greven et al., 2019).

Given that over 300 million people globally live with mental disorders (GBD 2017 Disease and Injury Incidence and Prevalence Collaborators, 2018), findings regarding the association between cognitive emotion regulation strategies and mental health can provide key information for practice. Although such associations may differ by the level of environmental sensitivity (Aldao et al., 2010; Yano et al., 2021a), few studies have examined their moderating effect. Therefore, further longitudinal studies are required to enhance their adaptability to practice (Yano and Oishi, 2018).

A popular statistical method for longitudinal data is latent growth model analysis (Duncan et al., 2013), as it can describe individuals’ behavior or status, in terms of their initial levels and change rates over time. Additionally, the associations of the (changes in) predictors and their interaction terms (Klein and Moosbrugger, 2000) with the (changes in) outcomes can be evaluated using latent moderated structural equation modeling (LMS).

To overcome the limitations of past studies, this longitudinal study aimed to investigate whether environmental sensitivity moderates the associations between each strategy of cognitive emotion regulation and mental health. Building on the existing findings (Bratholm Wyller et al., 2017; Yano et al., 2021a; Lionetti et al., 2022), the authors hypothesized the moderating effect of environmental sensitivity on the associations between *positive reappraisal*, *rumination*, *catastrophizing*, and *refocusing on planning* and mental health: the former three strategies play a vital role for those with higher sensitivity and the other is important for low-sensitivity individuals. Unfortunately, clear hypotheses on other strategies could not be proposed because of a lack of evidence.

## 2 Materials and methods

### 2.1 Participants and procedures

This three-wave longitudinal study recruited Japanese university students from all prefectures through Cross Marketing, Inc.^3^ At Time 1 (November 2020), 1,233 students consented to participate in the study after they received an explanation of the study’s purpose and procedures. Data were subsequently collected from 585 and 349 university students at Time 2 (February 2021) and Time 3 (May 2021), respectively. The intervals between each survey were set so that the overall period was 6 months (Porru et al., 2022; Eisma et al., 2023).

The Directed Questions Scale (Maniaci and Rogge, 2014) was included in each scale to assess the respondents’ attitudes toward the survey. Based on Maniaci and Rogge (2014) recommendation, in each survey, the responses were treated as missing values when a participant did not follow the Directed Questions Scale more than *M* + 2.7 *SD* times (i.e., thrice at Time 1, twice at Time 2, and once at Time 3). The number of participants who provided data and were included in the analyses is shown in Table 1. The comparison of the characteristics between the participants who completed all the surveys and those who did not indicated that while more women completed the surveys, men were likely to drop out (ꭓ^2^(2) = 16.69, *p* < 0.001, Cramer’s *V* = 0.12). Moreover, the complete group was older than the incomplete group (*t*(642.18) = 3.30, *p* = 0.001, Cohen’s *d* = 0.21). Among the psychological characteristics, there were insignificant or negligible (though significant) differences (for the details, see Supplementary Table S1).

**Table 1:** Characteristics of the participants in this study.

	Measurement Timepoint		
	Time 1	Time 2	Time 3
Gender (*n*)			
Men	566 (540)	230 (221)	129 (116)
Women	650 (629)	343 (335)	215 (203)
Other/unidentified	17 (17)	12 (12)	6 (6)
Mean age (*SD*)	20.2 (1.4)	NA	NA

Values in the brackets indicate the number of participants used in the analyses. NA, Not available because their age was not provided at Time 2 and Time 3.

The study’s procedures were approved by the Ethics Committee of Rikkyo University (Nos. KOMI19001A and KOMI20010A).

### 2.2 Measurements

#### 2.2.1 Environmental sensitivity

This study considered the concept of SPS as a marker of environmental sensitivity (Slagt et al., 2018; Greven et al., 2019) and assessed it using the 10-item Japanese version of the HSP scale (HSP-J10) (Iimura et al., 2023)—a shorter version of the original HSP scale (Aron and Aron, 1997). The HSP-J10 has a bi-factor structure with a general sensitivity factor, in addition to Ease of Excitation (five items), Low Sensory Threshold (three items), and Aesthetic Sensitivity (two items). Each item was rated on a seven-point Likert-type scale (1 = “*Strongly disagree*” to 7 = “*Strongly agree*”), with higher scores indicating higher SPS. As mentioned in the Introduction, SPS is a heritable trait (Assary et al., 2021) and the scores of the HSP scale have high temporal stability (Konrad and Herzberg, 2019; Iimura et al., 2023). Therefore, the authors assessed the HSP-J10 only at Time 1.

#### 2.2.2 Mental health

The Japanese version of the Kessler 10 scale (Kessler et al., 2002; Furukawa et al., 2008) was used to assess mental health at all the measurement points. This scale consists of 10 items, which ask respondents how often they experienced depressive or anxiety symptoms during the last month. Each item was rated on a five-point Likert-type scale (0 = “*None of the time*” to 4 = “*all of the time*”), with higher scores indicating poorer mental health.

#### 2.2.3 Cognitive emotion regulation

The Japanese version of the CERQ-short (Garnefski and Kraaij, 2006a; Sakakibara, 2017) was used at all the measurement points to assess how often the participants employed the nine conceptually distinct strategies to regulate their emotions in a general and particular situation, with two items in each strategy. Each item was rated on a five-point Likert-type scale (1 = “*[Almost] never*” to 5 = “*[Almost] always*”), with higher scores indicating more frequent use of a specific strategy. This being the first study to investigate the moderating role of sensitivity in the association between cognitive strategies and mental health, the authors decided to focus on a general situation.

#### 2.2.4 Neuroticism

Given that some items of the HSP-J10 involved negative words such as “uncomfortable” and “overwhelmed,” despite the neutrality of environmental sensitivity, it could be negatively biased to correlate with other psychological concepts (Greven et al., 2019). Therefore, in line with previous studies (Yano et al., 2021b), the current study included neuroticism as a control variable. The participants also responded to two items from the Japanese version of the 10-Item Personality Inventory (Gosling et al., 2003; Oshio et al., 2012) only at Time 1. Each item was rated on a seven-point Likert-type scale (1 = “*Strongly disagree*” to 7 = “*Strongly agree*”), with higher scores indicating higher neuroticism. As the two-item correlation was weak but significant (*r* = 0.11, *p* < 0.001), the authors considered its reliability to be acceptable.

### 2.3 Statistical analyses

**Figure 1:**
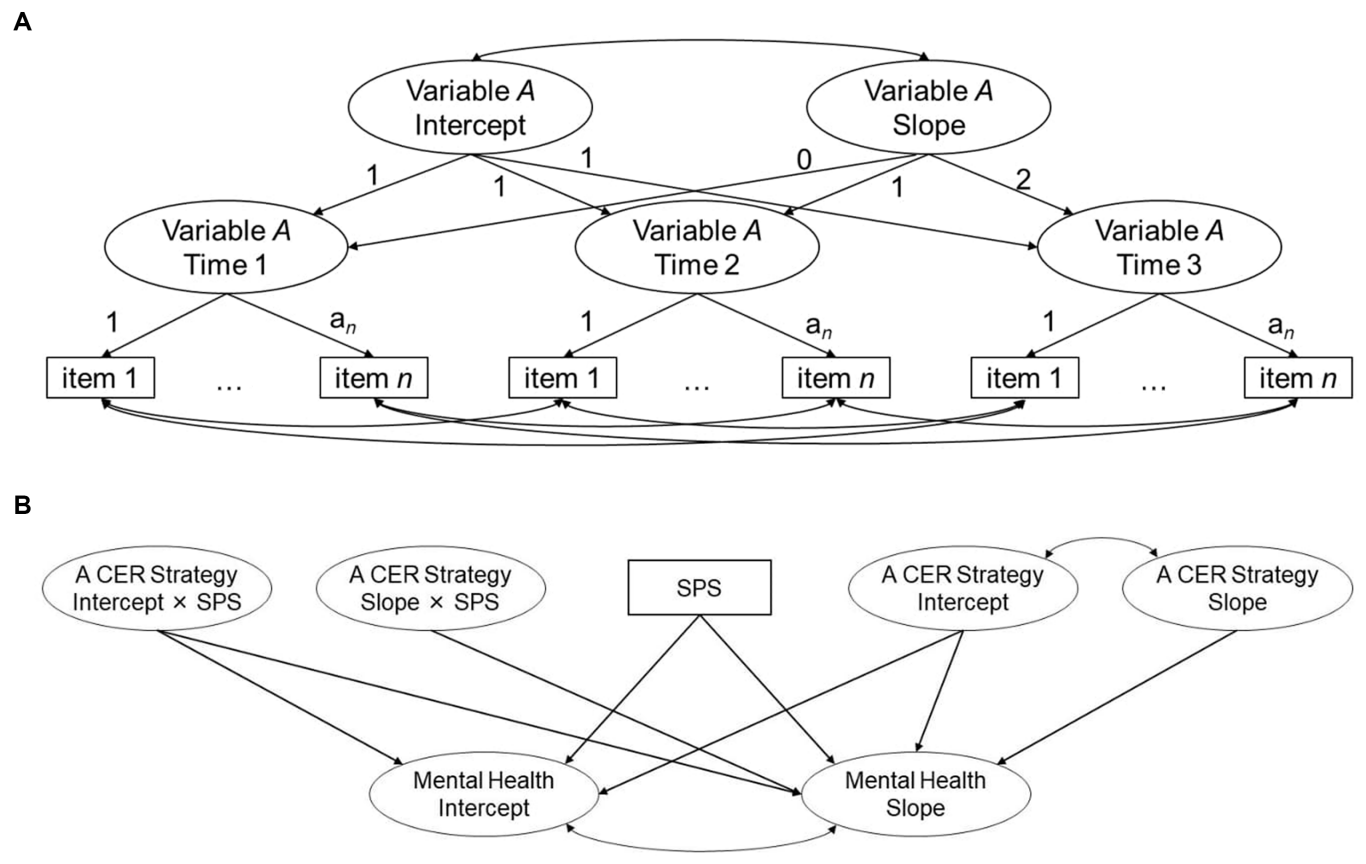
Illustration of the models estimated in this study. CER, Cognitive Emotion Regulation; SPS, Sensory Processing Sensitivity. Panel **(A)** illustrates a univariate latent growth model in which mental health or a cognitive emotion regulation strategy was modeled. Panel **(B)** represents a latent moderated structural equation model in which control variables (i.e., gender, age, and neuroticism) predicting the intercept and slope of mental health were omitted for brevity. When the multivariate latent growth model analysis was performed, the two interaction terms were not included in the model.

After investigating the correlations of the cognitive strategies with SPS and mental health, the authors performed three statistical analyses. First, second-order univariate latent growth model analyses (Hancock et al., 2001) were conducted to estimate the mean levels (i.e., intercept) and change rates (i.e., slopes) in mental health and each cognitive emotion regulation strategy (Figure 1A).^4^ To achieve metric equivalence in the latent constructs, unstandardized factor loadings and error variances were constrained equally between the measurement points. Second, the associations between SPS and each strategy with mental health were investigated using a multivariate latent growth model, followed by LMS analyses in which the interaction terms between SPS and the intercept and slope of a cognitive strategy were added into the predictors (Figure 1B). All the models involved three control variables (gender, age, and neuroticism) and were estimated using the robust maximum likelihood estimation method. The Mplus code required for the LMS analysis was taken from Maslowsky et al. (2015). Finally, the models with and without interaction terms were compared through log-likelihood ratio difference tests (Maslowsky et al., 2015), using an appropriate correction for the maximum likelihood estimation method (Satorra and Bentler, 2001). When the former model’s fit to data was better than the latter’s and the interaction terms were significantly associated with the intercept and/or slope of mental health, the authors performed simple slope tests and calculated the region of significance for SPS.^5^

In the univariate and multivariate latent growth model analyses, the goodness of fit of the model was comprehensively evaluated based on the comparative fit index (CFI) and root mean square error of approximation (RMSEA). Values >0.90 for CFI and < 0.10 for RMSEA were acceptable (Kline, 2005). Given that these analyses estimated a large number of parameters, to control for the inflation of Type I error rates, the false discovery rate procedure was employed (Benjamini and Hochberg, 1995). The authors used the free statistical software HAD ver. 17.20 (Shimizu, 2016) for the correlation analysis; Mplus ver. 8.3 (Muthén and Muthén, 2017) for the latent growth model and LMS analyses; and a free application^6^ introduced by Roisman et al. (2012) for the simple slope tests and to calculate the regions of significance. The significance levels were set at *p* < 0.05, and given the large sample size, the effect size was also considered to interpret the correlations (i.e., 0.10, 0.20, and 0.30 as relatively small, typical, and relatively large effect sizes, respectively; Gignac and Szodorai, 2016). Missing values were handled using the full information maximum likelihood estimation method.

## 3 Results

### 3.1 Preliminary analyses

For the preliminary analyses, the correlation coefficients between SPS, mental health, and the cognitive emotion regulation strategies were calculated (Table 2). The results indicated that SPS was positively correlated with *rumination* (relatively large), *self-blame* (typical), and *catastrophizing* (typical to relatively large) measured at all the time points as well as with *acceptance* (relatively small to typical) and *refocusing on planning* (relatively small) at Time 1 and Time 2 (*p* < 0.05). Significant correlations were found between mental health and *positive reappraisal*, *self-blame*, *positive refocusing* (relatively small to typical), *rumination*, and *catastrophizing* (typical to relatively large), at all the corresponding time points; *putting into perspective* at Time 2 and Time 3 (relatively small); and *refocusing on planning* (relatively small) only at Time 2 (*p* < 0.05). Additionally, the rank-order stabilities for all the nine strategies were greater than “relatively large” (*r* > 0.40; see Supplementary Table S3).

**Table 2:** Descriptive statistics and correlations between the variables.

		SPS	Mental Health			Mean (*SD*)	ɑ
			Time 1	Time 2	Time 3		
Positive Reappraisal	Time 1	0.01	−0.14***	−0.10*	−0.10	3.26 (0.97)	0.70
	Time 2	−0.05	−0.24***	−0.20***	−0.27***	3.26 (0.99)	0.75
	Time 3	−0.03	−0.22***	−0.17**	−0.19**	3.29 (0.99)	0.75
Putting into Perspective	Time 1	0.07*	−0.08**	−0.11*	−0.13*	3.16 (0.93)	0.57
	Time 2	−0.05	−0.20***	−0.18***	−0.17**	3.13 (0.93)	0.65
	Time 3	−0.04	−0.21***	−0.18**	−0.17**	3.11 (0.91)	0.57
Rumination	Time 1	0.42***	0.28***	0.29***	0.30***	3.42 (0.93)	0.59
	Time 2	0.33***	0.25***	0.27***	0.26***	3.46 (0.89)	0.59
	Time 3	0.35***	0.23***	0.26***	0.34***	3.34 (0.93)	0.67
Acceptance	Time 1	0.20***	0.02	−0.000	0.04	3.54 (0.89)	0.77
	Time 2	0.11*	−0.07	−0.05	−0.11*	3.50 (0.91)	0.80
	Time 3	0.07	−0.17**	−0.09	−0.02	3.48 (0.94)	0.82
Self-Blame	Time 1	0.28***	0.21***	0.17***	0.20***	3.34 (0.92)	0.73
	Time 2	0.25***	0.17***	0.19***	0.18**	3.34 (0.90)	0.78
	Time 3	0.23***	0.08	0.12*	0.21***	3.35 (0.93)	0.82
Positive Refocusing	Time 1	−0.01	−0.10***	−0.16***	−0.20***	3.05 (0.93)	0.70
	Time 2	−0.06	−0.18***	−0.15***	−0.18**	3.08 (0.95)	0.79
	Time 3	−0.10	−0.24***	−0.27***	−0.21***	3.04 (0.93)	0.78
Blaming Others	Time 1	0.06*	0.07*	0.04	−0.02	2.79 (0.85)	0.68
	Time 2	−0.07	−0.06	−0.04	−0.10	2.82 (0.84)	0.72
	Time 3	0.04	−0.08	−0.07	−0.02	2.75 (0.89)	0.81
Catastrophizing	Time 1	0.34***	0.35***	0.34***	0.34***	3.03 (0.97)	0.75
	Time 2	0.32***	0.25***	0.29***	0.26***	3.13 (0.97)	0.78
	Time 3	0.26***	0.21***	0.24***	0.31***	3.00 (0.96)	0.78
Refocusing on Planning	Time 1	0.18***	−0.06*	−0.06	−0.04	3.51 (0.91)	0.78
	Time 2	0.09*	−0.16***	−0.12**	−0.14**	3.43 (0.89)	0.75
	Time 3	0.10	−0.14*	−0.11	−0.10	3.44 (0.90)	0.82
Mean (*SD*)		4.22 (1.10)	1.38 (0.99)	1.30 (1.03)	1.36 (1.07)		
ɑ		0.87	0.94	0.95	0.95		

SPS, Sensory Processing Sensitivity.

*p < 0.05.

**p < 0.01.

***p < 0.001.

### 3.2 Change in mental health and cognitive emotion regulation strategies

To estimate the initial level and change rate in each variable, a series of univariate latent growth model analyses were conducted (Table 3). The covariances were set between some items measured at the same time points based on the modification indices (Slagt et al., 2018). While the fit indices were acceptable for all the models (CFI = 0.90–1.00, RMSEA = 0.00–0.07), for *acceptance*, only the initial level and its variance were estimated owing to the negative variance of its slope factor. The variances of the slope factors in the other strategies and mental health showed significant values (*p* < 0.001), indicating that there were significant inter-individual differences in the variables’ change rates. The scores of *refocusing on planning* decreased over the time points at the mean level (*p* = 0.009). Furthermore, negative correlations were seen between the intercept and slope in all the variables, except *acceptance* (*p* < 0.01).

**Table 3:** Estimated parameters of the univariate latent growth models.

	Intercept		Slope		*r*
	Mean (SE)	Variance (SE)	Mean (SE)	Variance (SE)	
Mental Health	1.57*** (0.03)	1.15*** (0.04)	0.02 (0.02)	0.24*** (0.03)	−0.35***
Positive Reappraisal	3.30*** (0.03)	0.69*** (0.04)	0.01 (0.02)	0.10*** (0.02)	−0.22**
Putting into Perspective	3.15*** (0.03)	0.48*** (0.04)	−0.02 (0.02)	0.09*** (0.02)	−0.46***
Rumination	3.38*** (0.03)	0.48*** (0.04)	−0.02 (0.02)	0.12*** (0.02)	−0.42***
Acceptance	3.54*** (0.03)	0.42*** (0.04)	NA	NA	NA
Self-Blame	3.44*** (0.03)	0.63*** (0.04)	−0.002 (0.02)	0.09** (0.03)	−0.39***
Positive Refocusing	3.05*** (0.03)	0.58*** (0.04)	−0.001 (0.02)	0.18*** (0.03)	−0.51***
Blaming Others	2.78*** (0.03)	0.46*** (0.03)	−0.02 (0.02)	0.10*** (0.03)	−0.49***
Catastrophizing	3.03*** (0.03)	0.66*** (0.04)	0.004 (0.02)	0.14*** (0.02)	−0.47***
Refocusing on Planning	3.49*** (0.03)	0.61*** (0.04)	−0.05** (0.02)	0.11*** (0.02)	−0.35***

SE, Standard Error. NA, The slope factor was not included in the model because of its negative variance.

**p < 0.01.

***p < 0.001.

### 3.3 Associations between mental health and cognitive emotion regulation strategies

First, nine models were estimated using a multivariate latent growth model, with the intercept and slope of the cognitive strategy, SPS, and control variables (i.e., gender, age, and neuroticism) as predictors and the intercept and slope of mental health as outcomes. The results showed good fit indices for all the models (CFI = 0.91–0.93, RMSEA = 0.04). Next, we estimated nine LMS models in which the interaction terms between SPS and the intercept or slope of the cognitive strategies were added (Figure 1B). A series of log-likelihood ratio difference tests suggested that the models with interactions fitted the data better when *putting into perspective*, *rumination*, *blaming others*, *catastrophizing*, or *refocusing on planning* were predictors, whereas those without interactions were supported when *positive reappraisal*, *acceptance*, *self-blame*, or *positive refocusing* were predictors (Table 4).

**Table 4:** Comparing the models with and without interactions.

	Positive Reappraisal		Putting into Perspective		Rumination	
	Without Interaction	With Interaction	Without Interaction	With Interaction	Without Interaction	With Interaction
Log-likelihood	−28183.14	−28180.08	−28488.89	−28393.53	−28298.21	−28288.44
No. Parameters	115	118	116	119	115	118
Δ Log-likelihood	χ^2^(3) = 6.13, *p* = 0.20		χ^2^(3) = 190.72, *p* < 0.001		χ^2^(3) = 19.54, *p* < 0.001	
	Acceptance		Self-Blame		Positive Refocusing	
	Without Interaction	With Interaction	Without Interaction	With Interaction	Without Interaction	With Interaction
Log-likelihood	−27628.82	−27626.72	−27855.26	−27851.50	−28055.59	−28051.57
No. Parameters	114	116	116	119	116	119
Δ Log-likelihood	χ^2^(2) = 4.20, *p* = 0.25		χ^2^(3) = 7.53, *p* = 0.09		χ^2^(3) = 8.03, *p* = 0.08	
	Blaming Others		Catastrophizing		Refocusing on Planning	
	Without Interaction	With Interaction	Without Interaction	With Interaction	Without Interaction	With Interaction
Log-likelihood	−27771.09	−27762.22	−28050.87	−28043.83	−27778.78	−27773.55
No. Parameters	117	120	116	119	115	118
Δ Log-likelihood	χ^2^(3) = 17.73, *p* < 0.001		χ^2^(3) = 14.08, *p* = 0.002		χ^2^(3) = 10.46, *p* = 0.02	

The models in bold type were used in the subsequent analyses.

The estimated parameters for the final model are presented in Table 5. To save space, the results for the control variables are in the Supplementary materials (see Supplementary Tables S4–S12). In the four models without interactions, the intercepts of *positive reappraisal*, *acceptance*, and *positive refocusing* were negatively associated with the intercept of mental health (*p* < 0.01), whereas *self-blame* was not significantly associated. In the five models with interactions, the intercepts of *putting into perspective* and *refocusing on planning* were negatively associated with mental health (*p* < 0.001). Additionally, positive associations were indicated between the intercepts of *rumination* and *catastrophizing* and mental health and between the slopes of those strategies and mental health (*p* < 0.01). Furthermore, (changes in) mental health was significantly associated with the five interaction terms between SPS and the intercepts of *rumination* and *blaming others*, and the slopes of *blaming others*, *catastrophizing*, and *refocusing on planning* (*p* < 0.05). Finally, SPS was positively associated with the intercept of mental health (*p* < 0.001).

**Table 5:** Associations of the cognitive emotion regulation strategies and SPS with mental health.

	Positive Reappraisal			Putting into Perspective			Rumination		
	*b*	*b SE*	β	*b*	*b SE*	β	*b*	*b SE*	β
SPS = > I Mental Health	0.04	0.003	0.40***	0.04	0.003	0.39***	0.03	0.003	0.35***
I CER = > I Mental Health	−0.21	0.04	−0.17***	−0.24	0.05	−0.17***	0.22	0.05	0.16**
I CER × SPS = > I Mental Health		NA		−0.004	0.004	−0.03	0.01	0.004	0.10**
SPS = > S Mental Health	−0.001	0.003	0.04	−0.002	0.003	−0.05	−0.003	0.003	−0.07
I CER = > S Mental Health	0.01	0.04	0.02	0.001	0.05	0.002	0.06	0.05	0.10
S CER = > S Mental Health	−0.37	0.19	−0.22*	−0.007	0.17	−0.005	0.43	0.12	0.35***
I CER × SPS = > S Mental Health		NA		−0.006	0.004	−0.11	−0.008	0.004	−0.14*
S CER × SPS = > S Mental Health		NA		−0.03	0.01	−0.22*	−0.02	0.01	−0.18*
	Acceptance			Self-Blame			Positive Refocusing		
	*B*	*b SE*	β	*b*	*b SE*	β	*b*	*b SE*	β
SPS = > I Mental Health	0.04	0.003	0.39***	0.04	0.003	0.37***	0.04	0.003	0.38***
I CER = > I Mental Health	−0.20	0.06	−0.12**	0.11	0.05	0.08*	−0.16	0.05	−0.12**
I CER × SPS = > I Mental Health		NA			NA			NA	
SPS = > S Mental Health	−0.002	0.03	−0.04	−0.002	0.003	−0.05	−0.001	0.003	−0.03
I CER = > S Mental Health	0.04	0.05	0.06	0.16	0.13	0.26	−0.03	0.05	−0.06
S CER = > S Mental Health		NA		1.67	1.71	0.56	−0.09	0.11	−0.08
I CER × SPS = > S Mental Health		NA			NA			NA	
S CER × SPS = > S Mental Health		NA			NA			NA	
	Blaming Others			Catastrophizing			Refocusing on Planning		
	*B*	*b SE*	β	*b*	*b SE*	β	*b*	*b SE*	β
SPS = > I Mental Health	0.04	0.003	0.38***	0.03	0.003	0.34***	0.04	0.003	0.41***
I CER = > I Mental Health	0.05	0.05	0.03	0.26	0.05	0.22***	−0.21	0.05	−0.15***
I CER × SPS = > I Mental Health	0.003	0.004	0.03	0.006	0.003	0.06	0.000	0.004	0.001
SPS = > S Mental Health	−0.004	0.003	−0.09	−0.004	0.003	−0.09	−0.003	0.003	−0.07
I CER = > S Mental Health	0.009	0.06	0.01	0.07	0.04	0.13	0.03	0.04	0.05
S CER = > S Mental Health	0.37	0.21	0.22	0.31	0.11	0.26**	−0.14	0.12	−0.10
I CER × SPS = > S Mental Health	−0.01	0.005	−0.21**	−0.006	0.003	−0.13	−0.006	0.004	−0.11
S CER × SPS = > S Mental Health	−0.06	0.02	−0.36***	−0.02	0.009	−0.21**	−0.02	0.008	−0.16*

SE, standard error; I, intercept; S, slope; SPS, Sensory Processing Sensitivity; CER, Cognitive Emotion Regulation. NA, not available because the paths were not estimated. For brevity, the paths from the control variables to mental health were omitted. The underlined values in italics are considered falsely positive when controlling for the inflation of type I error rates.

*p < 0.05.

**p < 0.01.

***p < 0.001.

**Figure 2:**
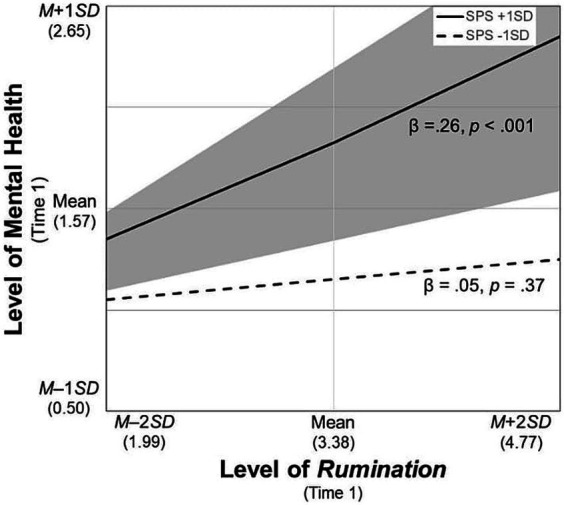
Simple slopes of *rumination* on mental health at Time 1. SPS, Sensory Processing Sensitivity. The shaded area indicates the SPS values at which the predictor is significantly associated with the level of mental health (i.e., lower than *M* – 4.38 *SD* and greater than *M* – 0.46 *SD*).

**Figure 3:**
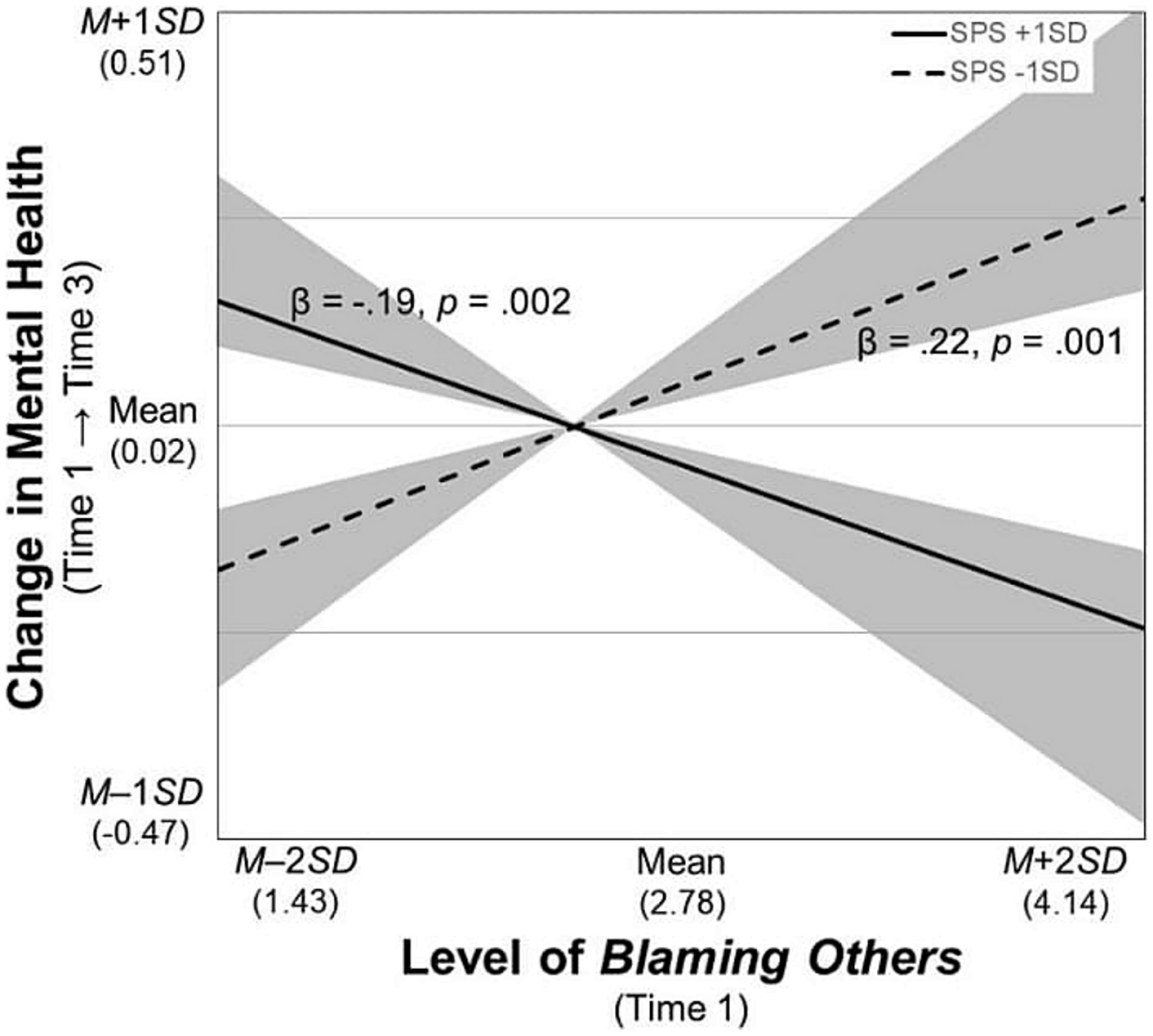
Simple slopes of *blaming others* at Time 1 on changes in mental health. SPS, Sensory Processing Sensitivity. The shaded area indicates the SPS values at which the predictor is significantly associated with the slopes of mental health (i.e., lower than *M* – 0.49 *SD* and greater than *M* + 0.61 *SD*).

**Figure 4:**
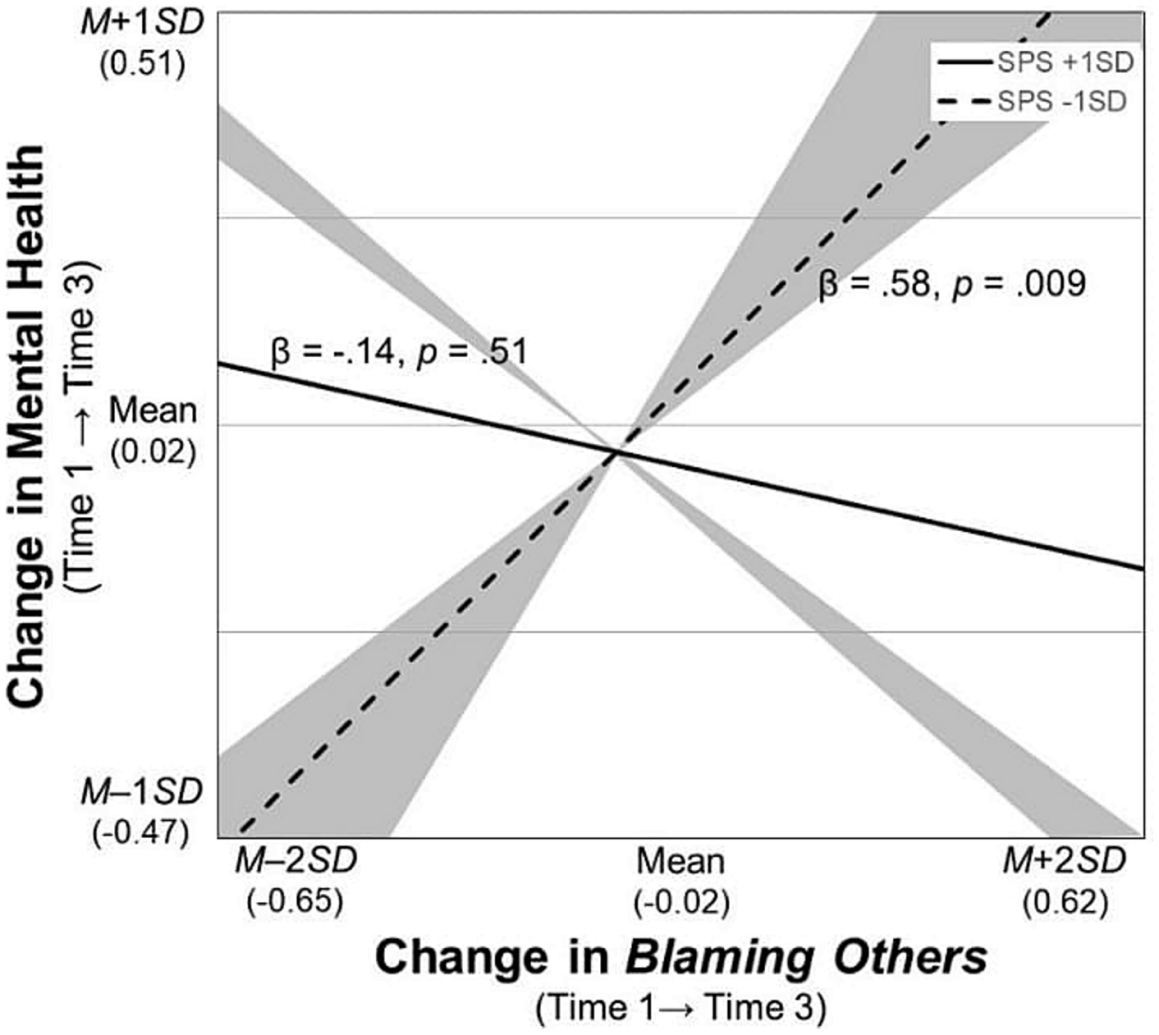
Simple slopes of change in *blaming others* on changes in mental health. *Notes*. SPS, Sensory Processing Sensitivity. The shaded area indicates the SPS values at which, the predictor is significantly associated with the slopes of mental health (i.e., lower than *M* – 0.58 *SD* and greater than *M* + 1.79 *SD*).

**Figure 5:**
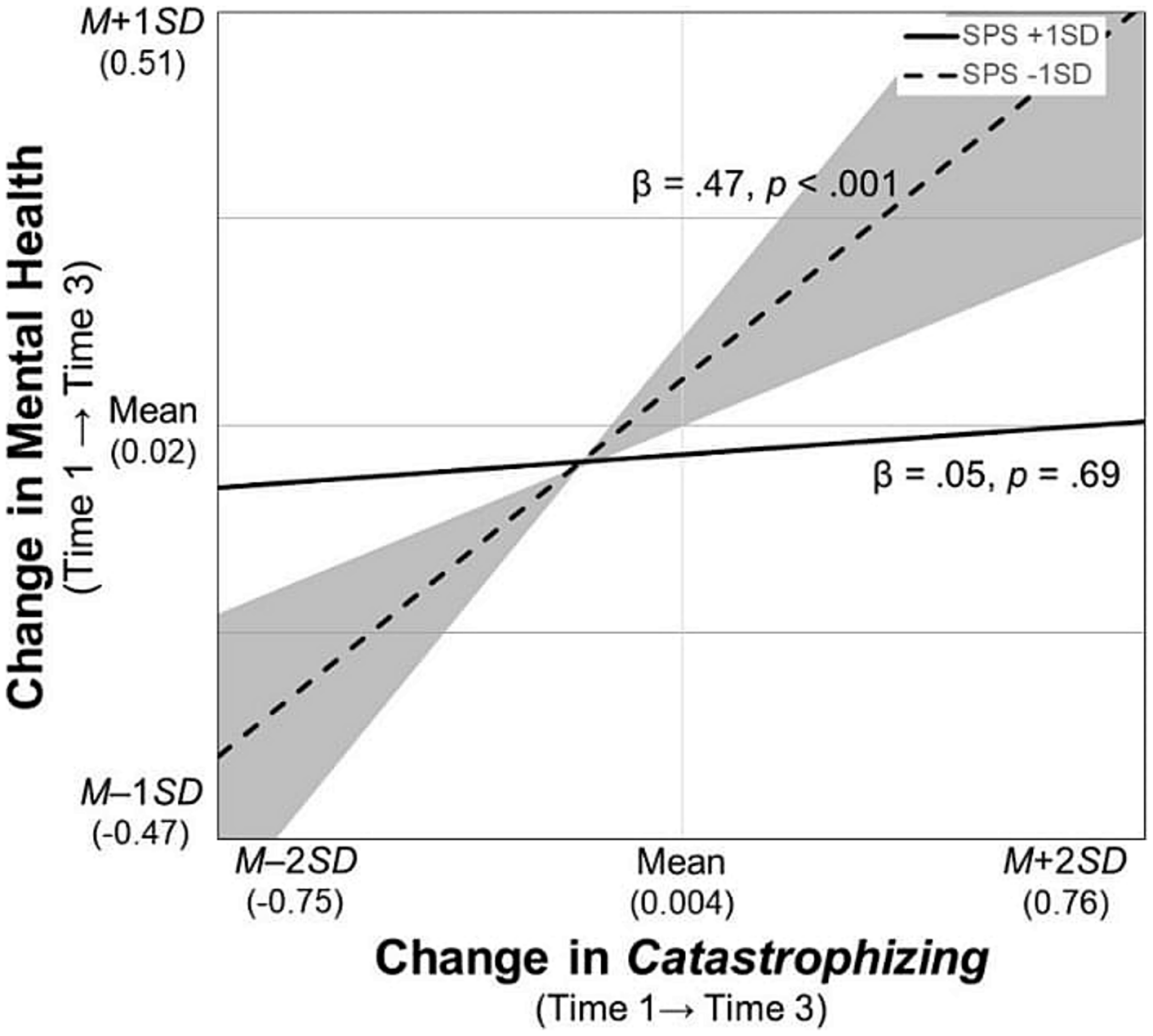
Simple slopes of change in *catastrophizing* on changes in mental health. SPS, Sensory Processing Sensitivity. The shaded area indicates the SPS values, at which, the predictor is significantly associated with the slopes of mental health (i.e., lower than *M* + 0.20 *SD* and greater than *M* + 2.51 *SD*).

**Figure 6:**
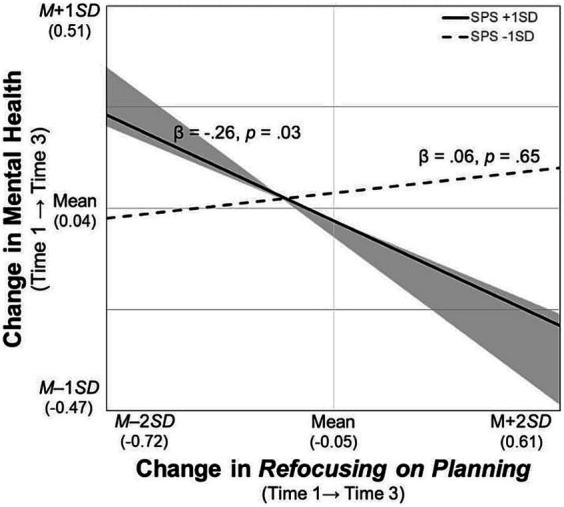
Simple slopes of change in *refocusing on planning* on changes in mental health. SPS, Sensory Processing Sensitivity. The shaded area indicates the SPS values, at which, the predictor is significantly associated with the slopes of mental health (i.e., lower than *M* – 2.47 *SD* and greater than *M* + 0.83 *SD*).

For the significant interaction terms, simple slope tests were conducted and the regions of significance of the moderator (i.e., SPS) were calculated. First, the participants with SPS scores higher than *M* – 0.46 *SD* had poorer mental health when they used *rumination* more frequently (Figure 2). Second, those who frequently used *blaming others* improved their mental health over time when their SPS scores were higher than *M* + 0.61 *SD*, whereas their mental health worsened when their SPS scores were lower than *M* – 0.49 *SD* (Figure 3). Third, likewise, the participants who increased their frequency of *blaming others* over time improved their mental health when their SPS scores were higher than *M* + 1.79 *SD*, whereas their mental health worsened with SPS scores lower than *M* – 0.58 *SD* (Figure 4). Fourth, the mental health of the participants whose frequent use of *catastrophizing* increased worsened further only when their SPS scores were lower than *M* + 0.20 *SD* (Figure 5). Finally, when the participants’ frequent use of *refocusing on planning* kept increasing, their mental health further improved only when their SPS scores were higher than *M* + 0.83 *SD* (Figure 6).

## 4 Discussion

### 4.1 Associations of the cognitive emotion regulation strategies with mental health

This study conducted a three-wave longitudinal survey and investigated the associations between (the changes in) the nine cognitive emotion regulation strategies and (the changes in) mental health based on individual differences in environmental sensitivity. The results indicated that the interactions between sensitivity and (the changes in) four of the strategies significantly predicted mental health at the initial level or its rate of change over time (Table 5).

First, more frequent use of *rumination* was associated with poorer mental health at Time 1 only for those whose sensitivity scores were higher than *M* – 0.46 *SD* (Figure 2). This result was consistent with our hypothesis and could elaborate the existing finding that highly sensitive individuals tend to engage in ruminative thinking in less supportive environments (Lionetti et al., 2022). Compared with individuals with low sensitivity, the effect of rumination on mental health may be stronger for highly sensitive individuals owing to their characteristics related to in-depth processing of internal information such as feelings and thoughts (Aron et al., 2012; Greven et al., 2019). Further, given the positive association between the slopes of these variables, regardless of their sensitivity level, an increase in *rumination* could be a risk factor even when individuals have low sensitivity.

While the significantly positive association between *catastrophizing* and mental health at Time 1 was consistent with previous findings (Garnefski and Kraaij, 2006a; Potthoff et al., 2016; Urano et al., 2022), their association in terms of the longitudinal data was inconsistent with the hypothesis. Rather than in high-sensitivity individuals, the change in *catastrophizing* may be an important risk factor for worsened mental health in lower-sensitivity individuals (i.e., scores lower than *M* + 0.20 *SD*) (Figure 5). The positive and relatively large correlation between sensitivity and *catastrophizing* at Time 1 (see Table 2) is a possible explanation for the insignificant association between the slopes of this strategy and mental health in highly sensitive individuals; that is, they might have little room for the strategy to increase over time.

In terms of the cross-sectional data, those participants who frequently used *refocusing on planning* were likely to have better mental health, which is consistent with previous findings (Potthoff et al., 2016; Urano et al., 2022). Contrary to the hypothesis, only when sensitivity was higher than *M* + 0.83 *SD* did those with an increase in using *refocusing on planning* tend to improve their mental health over time (Figure 6). This finding could provide novel insights into future research and practice because previous studies have suggested that problem-focused coping has negative consequences for highly sensitive individuals in some cases (Yano et al., 2021a) and that they could benefit more from an intervention program focusing on emotional coping skills (Pluess and Boniwell, 2015; Kibe et al., 2020). However, a significant association was not found between the slopes of *refocusing on planning* and mental health in low-sensitivity individuals, indicating that just increasing the frequency of thinking about how to resolve problems does not improve mental health. Given that decision-making skills such as summarizing information and planning play a vital role in alleviating depressive symptoms in individuals with low sensitivity (Yano et al., 2021b), future studies should consider the process through which this strategy could be linked to their behaviors.

The other hypothesis was also not supported. The strategy of *positive reappraisal*, as well as *putting into perspective*, *acceptance*, and *positive refocusing*, was associated with better mental health at Time 1, regardless of the sensitivity level, which was consistent with their theoretical backgrounds (Garnefski et al., 2001). However, in some cases, *acceptance* was correlated with more serious symptoms of depression and anxiety owing to this strategy’s scope being measured by the CERQ (−short), namely, a passive form of acceptance similar to resignation to negative experiences (Garnefski and Kraaij, 2006b; Urano et al., 2022). This study failed to replicate the association of *self-blame* with mental health, despite plenty of evidence for its contribution to psychological symptoms (Garnefski and Kraaij, 2006a, 2006b). This inconsistency could be explained by that the use of this strategy in the present study’s sample at Time 1 was more frequent than that in Garnefski and Kraaij (2006a) study where the sample responded to the CERQ-short (Cohen’s *d* = 1.39). However, the reason for our results remains questionable.

Finally, the more frequent use of *blaming others* at Time 1, or its increase over time, may worsen mental health when individuals have a sensitivity score lower than *M* – 0.49 *SD* or *M* – 0.58 *SD* (Figures 3, 4). These results advance the findings of the positive correlations between this strategy and depression or anxiety reported in cross-sectional research (Garnefski et al., 2001; Garnefski and Kraaij, 2006b; Potthoff et al., 2016; Urano et al., 2022). By contrast, when individuals are highly sensitive (scores higher than *M* + 0.61 *SD* or *M* + 1.79 *SD*), the initial level or the increase in this strategy could contribute to improving mental health over time. These findings are inconsistent with the existing evidence, and they suggest that this strategy is not always associated with poorer mental health, although it has been considered maladaptive *a priori* (Garnefski et al., 2001). The moderating effect of sensitivity on these associations could be explained by individual differences in behavior after attributing negative events to another person. For example, in-depth processing of a variety of information and pausing to check before taking actions—the core characteristics of highly sensitive individuals (Aron et al., 2012)—could prevent them from blaming others at a behavioral level. Conversely, given that individuals with low sensitivity often make decisions without using enough caution (Yano et al., 2021b), they may blame others at the behavioral level, resulting in loss of social support (Tennen and Affleck, 1990). However, it should be noted, that the aforementioned explanation cannot fully capture the mechanism through which *blaming others* contributes to an adaptive outcome, as this study did not measure the level of social support that the participants received.

### 4.2 Strengths, limitations, and future directions

This study’s main findings are as follows: (1) the strategy of *blaming others* has contrastive effects on changes in mental health by the level of environmental sensitivity; (2) the increase in *refocusing on planning* is associated with improved mental health over time only for highly sensitive individuals; (3) the strategies of *rumination* and *catastrophizing* were the most important risk factors for mental health problems, but had different effects by sensitivity level; and (4) *positive reappraisal*, *putting into perspective*, *acceptance*, and *positive refocusing* were associated with better mental health only at the beginning of the study. In short, the strengths of our results are that they reveal the individual differences in the effects of the cognitive strategies and provide evidence for the role of sensitivity in mental health research. The current findings may also provide useful information for practice. Combined with Assary et al.’s (2023) suggestion, assessment before practice enables support providers to design and implement intervention programs based on individual differences in sensitivity. For example, while the program for promoting *refocusing on planning* and preventing *rumination* could improve mental health in highly sensitive people, emphasizing the reduction in *blaming others* could be effective for low-sensitivity people.

Despite the aforementioned strengths, some limitations should also be acknowledged. The first is the extent to which our findings can be generalized. As previous studies have suggested that age (Garnefski and Kraaij, 2006b) and cultural factors (Potthoff et al., 2016) moderate the association between these strategies and depression or anxiety, future studies should adopt a sample that excludes Japanese university students. Furthermore, contextual factors also moderate such associations, as mentioned above (e.g., Garnefski et al., 2005; Aldao et al., 2010). While this study focused on the use of each strategy in general, it is unclear which situations our findings could be generalized to, such as final semester exams and job hunting. Second, the measurement of the cognitive emotion regulation strategies should be improved. Given the respondents’ burden, this three-wave longitudinal study used the CERQ-short (Garnefski and Kraaij, 2006a; Sakakibara, 2017) that has acceptable but lower reliability than the original version (Garnefski and Kraaij, 2006a). Considering that a recent study revised the Japanese version of the CERQ and improved its psychometric properties (Urano et al., 2022), replicating this study using the revised scale could be useful for examining the robustness of this study’s findings. Finally, as this study focused on the cognitive aspects of emotion regulation strategies, it could not investigate the mechanisms through which each cognitive strategy was linked to what behavioral strategies and (mal) adaptive outcomes. Therefore, it is necessary to clarify this mechanism, as it is expected to provide key information for designing effective intervention programs. Further investigations should reveal the adaptive process of emotion regulation based on individual differences in sensitivity considering the framework of behavioral emotion regulation, which was recently proposed by Kraaij and Garnefski (2019).

## Data availability statement

The data analyzed in this study and Mplus code can be found in the Open Science Framework [https://osf.io/jymqk/?view_only=899e7768fc21410eb959fbfaa6ca7c17].

## Ethics statement

The studies involving humans were approved by College of Community and Human Services, Rikkyo University. The studies were conducted in accordance with the local legislation and institutional requirements. The participants provided their written informed consent to participate in this study.

## Author contributions

KY: Conceptualization, Formal analysis, Investigation, Writing – original draft, Writing – review & editing. KO: Investigation, Supervision, Writing – review & editing.

## Footnotes

^1^Pluess (2015) found that all the theories and concepts shared the perspective that people differ in sensitivity to both positive and negative influences; therefore, these were integrated into an umbrella framework.

^2^It should be noted that an empirical study failed to replicate the existing findings (Hellwig and Roth, 2021).

^3^Cross Marketing Inc. is one of the largest academic survey companies in Japan. At the time of the first survey (November 2020), they had about 5.4 million web survey monitors all over Japan. Many researchers have collaborated with the company to collect data (Yano et al., 2021a), suggesting the high quality and reliability of their data. The respondents were given points exchangeable for cash as rewards if they completed a questionnaire online.

^4^Some variables (e.g., *putting into perspective*) did not have sufficient reliability in this study. Thus, the authors decided to employ this method to control measurement errors as far as possible. Additionally, as a preliminary analysis, the authors examined the change pattern in each variable, namely, the nonlinear model vs. the linear model (Slagt et al., 2018). The log-likelihood ratio difference tests indicated that the fit of the nonlinear model, in which the Time 2 loading from the slope factor was freely estimated, did not significantly increase from that in the linear model, in which the loading was constrained to 1, for most of the variables (see Supplementary Table S2). Therefore, the authors decided to analyze the variables assuming their linear changes.

^5^The results of the simple slope analyses were interpreted when the region of significance on SPS was from −2 *SD* to +2 *SD* in which most of the respondents existed.

^6^https://www.yourpersonality.net/interaction/ros.pl.
