# Supplementary material for: Individual differences in environmental sensitivity: associations between cognitive emotion regulation and mental health
Source: Front Psychol. 2024 Mar 8;15:1322544. doi: 10.3389/fpsyg.2024.1322544 (PMC10957743; doi:10.3389/fpsyg.2024.1322544)
Supplement: Supplementary file 1 [file Data_Sheet_1.docx]

Supplementary Materials

Table S1. Welch’s *t*-tests of each variable at Time 1 between the participants who completed the surveys and those who did not

|  | Mean (*SD*) | | *t*(*df*) | Cohen’s *d* |
| --- | --- | --- | --- | --- |
|  | Complete Group (*n* = 342) | Incomplete Group (*n* = 844) |  |  |
| SPS | 4.21 (1.09) | 4.22 (1.11) | *t*(639.46) = 0.19 | 0.01 |
| Neuroticism | 4.22 (1.20) | 4.34 (1.18) | *t*(608.44) = 1.48 | 0.10 |
| Mental Health | 1.25 (1.01) | 1.43 (0.97) | *t*(612.36) = 2.94 | 0.19 |
| Positive Reappraisal | 3.26 (0.91) | 3.26 (1.00) | *t*(692.06) = 0.03 | 0.002 |
| Putting into Perspective | 3.17 (0.88) | 3.15 (0.96) | *t*(682.73) = 0.28 | 0.02 |
| Rumination | 3.37 (0.84) | 3.44 (0.96) | *t*(718.01) = 1.27 | 0.08 |
| Acceptance | 3.53 (0.81) | 3.55 (0.92) | *t*(713.05) = 0.38 | 0.02 |
| Self-Blame | 3.29 (0.88) | 3.36 (0.93) | *t*(663.19) = 1.22 | 0.08 |
| Positive Refocusing | 3.09 (0.83) | 3.03 (0.96) | *t*(722.76) = 0.92 | 0.06 |
| Blaming Others | 2.81 (0.80) | 2.78 (0.87) | *t*(684.51) = 0.73 | 0.05 |
| Catastrophizing | 3.02 (0.94) | 3.04 (0.98) | *t*(660.74) = 0.23 | 0.02 |
| Refocusing on Planning | 3.51 (0.81) | 3.51 (0.95) | *t*(728.56) = 0.03 | 0.002 |
| *Notes.* SPS = Sensory Processing Sensitivity. | | | | |

Table S2. Comparing the models assuming nonlinear and linear change patterns

|  | Mental Health | |  | Positive Reappraisal | |  | Putting into Perspective | |  | Rumination | |  | Acceptance | |
| --- | --- | --- | --- | --- | --- | --- | --- | --- | --- | --- | --- | --- | --- | --- |
|  | Nonlinear | Linear |  | Nonlinear | Linear |  | Nonlinear | Linear |  | Nonlinear | Linear |  | Nonlinear | Linear |
| Log-likelihood | -24815.02 | -24815.28 |  | NA | -5728.64 |  | -5938.44 | -5938.46 |  | -5884.57 | -5887.03 |  | -5171.15 | -5172.81 |
| No. Parameters | 94 | 93 |  | NA | 14 |  | 16 | 15 |  | 15 | 14 |  | 18 | 17 |
| Δ Log-likelihood | χ^2^(1) = 0.52, *p* = .76 | |  | NA | |  | χ^2^(1) = 0.04, *p* = .88 | |  | χ^2^(1) = 4.91, *p* = .24 | |  | χ^2^(1) = 3.32, *p* = .14 | |
|  | Self-Blame | |  | Positive Refocusing | |  | Blaming Others | |  | Catastrophizing | |  | Refocusing on Planning | |
|  | Nonlinear | Linear |  | Nonlinear | Linear |  | Nonlinear | Linear |  | Nonlinear | Linear |  | Nonlinear | Linear |
| Log-likelihood | -5332.17 | -5361.52 |  | -5567.26 | -5567.36 |  | -5285.89 | -5288.77 |  | -5596.77 | -5596.84 |  | NA | -5261.10 |
| No. Parameters | 16 | 15 |  | 16 | 15 |  | 17 | 16 |  | 16 | 15 |  | NA | 14 |
| Δ Log-likelihood | χ^2^(1) = 58.69, *p* < .001 | |  | χ^2^(1) = 0.19, *p* = .81 | |  | χ^2^(1) = 5.76, *p* = .19 | |  | χ^2^(1) = 0.14, *p* = .84 | |  | NA | |
| *Notes.* NA = The values could not be computed because the error occurred when estimating the model. | | | | | | | | | | | | | | |

Table S3. Rank-order stability of each strategy in cognitive emotion regulation

|  | Rank-Order Stability (Time 1 to Time 2) | |  | Rank-Order Stability (Time 2 to Time 3) | |
| --- | --- | --- | --- | --- | --- |
|  | *r* | *p* |  | *r* | *p* |
| Positive Reappraisal | 0.60 | <.001 |  | 0.58 | <.001 |
| Putting into Perspective | 0.48 | <.001 |  | 0.50 | <.001 |
| Rumination | 0.43 | <.001 |  | 0.48 | <.001 |
| Acceptance | 0.49 | <.001 |  | 0.43 | <.001 |
| Self-Blame | 0.50 | <.001 |  | 0.52 | <.001 |
| Positive Refocusing | 0.43 | <.001 |  | 0.40 | <.001 |
| Blaming Others | 0.40 | <.001 |  | 0.46 | <.001 |
| Catastrophizing | 0.49 | <.001 |  | 0.46 | <.001 |
| Refocusing on Planning | 0.49 | <.001 |  | 0.48 | <.001 |

Table S4. Parameters estimated in the multivariate latent growth model and the latent moderated equation model with *positive reappraisal* as a predictor

|  | **Without Interaction Model** | | | | | |  | With Interaction Model | | | | | |
| --- | --- | --- | --- | --- | --- | --- | --- | --- | --- | --- | --- | --- | --- |
|  | *b* | *b SE* | β | β *SE* | 95%CI | *p* |  | *b* | *b SE* | β | β *SE* | 95%CI | *p* |
| Gender => I Mental Health | -0.19 | 0.06 | -0.09 | 0.03 | -0.15, -0.04 | .001 |  | -0.19 | 0.06 | -0.09 | 0.03 | -0.15, -0.04 | .001 |
| Age => I Mental Health | 0.03 | 0.02 | 0.05 | 0.03 | -0.009, 0.09 | .11 |  | 0.03 | 0.02 | 0.04 | 0.03 | -0.009, 0.09 | .11 |
| Neuroticism => I Mental Health | 0.11 | 0.02 | 0.24 | 0.03 | 0.18, 0.31 | <.001 |  | 0.11 | 0.02 | 0.24 | 0.03 | 0.18, 0.31 | <.001 |
| SPS => I Mental Health | 0.04 | 0.003 | 0.40 | 0.03 | 0.34, 0.46 | <.001 |  | 0.04 | 0.003 | 0.40 | 0.03 | 0.34, 0.46 | <.001 |
| I CER => I Mental Health | -0.21 | 0.04 | -0.17 | 0.03 | -0.24, -0.10 | <.001 |  | -0.21 | 0.04 | -0.17 | 0.04 | -0.24, -0.10 | <.001 |
| I CER × SPS => I Mental Health |  |  |  |  |  |  |  | -0.003 | 0.004 | -0.02 | 0.03 | -0.08, 0.04 | .48 |
| Gender => S Mental Health | 0.04 | 0.05 | 0.04 | 0.05 | -0.06, 0.14 | .38 |  | 0.04 | 0.05 | 0.04 | 0.05 | -0.06, 0.14 | .41 |
| Age => S Mental Health | 0.01 | 0.02 | 0.03 | 0.05 | -0.06, 0.13 | .38 |  | 0.01 | 0.02 | 0.03 | 0.05 | -0.06, 0.13 | .50 |
| Neuroticism => S Mental Health | -0.02 | 0.01 | -0.10 | 0.06 | -0.22, 0.02 | .09 |  | -0.02 | 0.01 | -0.11 | 0.06 | -0.22, 0.009 | .07 |
| SPS => S Mental Health | -0.001 | 0.003 | -0.03 | 0.07 | -0.16, 0.09 | .61 |  | -0.002 | 0.003 | -0.04 | 0.06 | -0.16, 0.08 | .52 |
| I CER => S Mental Health | 0.01 | 0.04 | 0.02 | 0.07 | -0.11, 0.15 | .77 |  | 0.01 | 0.04 | 0.03 | 0.07 | -0.11, 0.16 | .72 |
| S CER => S Mental Health | -0.37 | 0.19 | -0.22 | 0.11 | -0.43, -0.02 | .03 |  | -0.34 | 0.19 | -0.20 | 0.11 | -0.42, 0.02 | .07 |
| I CER × SPS => S Mental Health |  |  |  |  |  |  |  | -0.004 | 0.004 | -0.08 | 0.07 | -0.23, 0.06 | .27 |
| S CER × SPS => S Mental Health |  |  |  |  |  |  |  | -0.02 | 0.02 | -0.12 | 0.10 | -0.31, 0.07 | .22 |
| *Notes.* SE = standard error, I = intercept, S = slope, SPS = Sensory Processing Sensitivity, CER = Cognitive Emotion Regulation. Gender was used as a dummy variable (1 = men, 2 =women, 3 =unidentified/other). The models in bold type were fitted better to data in this study. | | | | | | | | | | | | | |

Table S5. Parameters estimated in the multivariate latent growth model and the latent moderated equation model with *putting into perspective* as a predictor

|  | Without Interaction Model | | | | | |  | **With Interaction Model** | | | | | |
| --- | --- | --- | --- | --- | --- | --- | --- | --- | --- | --- | --- | --- | --- |
|  | *b* | *b SE* | β | β *SE* | 95%CI | *p* |  | *b* | *b SE* | β | β *SE* | 95%CI | *p* |
| Gender => I Mental Health | -0.18 | 0.06 | -0.09 | 0.03 | -0.14, -0.04 | .001 |  | -0.18 | 0.06 | -0.09 | 0.03 | -0.14, -0.04 | .001 |
| Age => I Mental Health | 0.03 | 0.02 | 0.04 | 0.03 | -0.02, 0.09 | .167 |  | 0.03 | 0.02 | 0.04 | 0.03 | -0.01, 0.09 | .16 |
| Neuroticism => I Mental Health | 0.11 | 0.02 | 0.25 | 0.03 | 0.19, 0.32 | <.001 |  | 0.12 | 0.02 | 0.25 | 0.03 | 0.19, 0.32 | <.001 |
| SPS => I Mental Health | 0.04 | 0.003 | 0.29 | 0.03 | 0.33, 0.46 | <.001 |  | 0.04 | 0.003 | 0.39 | 0.03 | 0.33, 0.45 | <.001 |
| I CER => I Mental Health | -0.25 | 0.05 | -0.18 | 0.04 | -0.25, -0.10 | <.001 |  | -0.24 | 0.05 | -0.17 | 0.04 | -0.25, -0.10 | <.001 |
| I CER × SPS => I Mental Health |  |  |  |  |  |  |  | -0.004 | 0.004 | -0.03 | 0.03 | -0.10, 0.03 | .30 |
| Gender => S Mental Health | 0.07 | 0.06 | 0.06 | 0.05 | -0.04, 0.16 | .24 |  | 0.03 | 0.05 | 0.03 | 0.05 | -0.07, 0.13 | .60 |
| Age => S Mental Health | 0.02 | 0.02 | 0.05 | 0.05 | -0.05, 0.14 | .32 |  | 0.01 | 0.02 | 0.03 | 0.05 | -0.06, 0.13 | .52 |
| Neuroticism => S Mental Health | -0.03 | 0.02 | -0.10 | 0.06 | -0.22, 0.02 | .09 |  | -0.02 | 0.01 | -0.10 | 0.06 | -0.21, 0.02 | .10 |
| SPS => S Mental Health | -0.002 | 0.004 | -0.04 | 0.06 | -0.16, 0.09 | .59 |  | -0.002 | 0.003 | -0.05 | 0.06 | -0.17, 0.07 | .39 |
| I CER => S Mental Health | 0.003 | 0.06 | 0.003 | 0.07 | -0.14, 0.15 | .96 |  | 0.001 | 0.05 | 0.002 | 0.08 | -0.16, 0.16 | .98 |
| S CER => S Mental Health | -0.08 | 0.22 | -0.04 | 0.11 | -0.26, 0.18 | .70 |  | -0.007 | 0.17 | -0.005 | 0.12 | -0.24, 0.23 | .97 |
| I CER × SPS => S Mental Health |  |  |  |  |  |  |  | -0.006 | 0.004 | -0.11 | 0.08 | -0.26, 0.04 | .14 |
| S CER × SPS => S Mental Health |  |  |  |  |  |  |  | -0.03 | 0.01 | -0.23 | 0.10 | -0.42, -0.03 | .02 |
| *Notes.* SE = standard error, I = intercept, S = slope, SPS = Sensory Processing Sensitivity, CER = Cognitive Emotion Regulation. Gender was used as a dummy variable (1 = men, 2 =women, 3 =unidentified/other). The models in bold type were fitted better to data in this study. | | | | | | | | | | | | | |

Table S6. Parameters estimated in the multivariate latent growth model and the latent moderated equation model with *rumination* as a predictor

|  | Without Interaction Model | | | | | |  | **With Interaction Model** | | | | | |
| --- | --- | --- | --- | --- | --- | --- | --- | --- | --- | --- | --- | --- | --- |
|  | *b* | *b SE* | β | β *SE* | 95%CI | *p* |  | *b* | *b SE* | β | β *SE* | 95%CI | *p* |
| Gender => I Mental Health | -0.18 | 0.06 | -0.09 | 0.03 | -0.14, -0.03 | .001 |  | -0.18 | 0.06 | -0.09 | 0.03 | -0.15, -0.04 | .001 |
| Age => I Mental Health | 0.03 | 0.02 | 0.04 | 0.03 | -0.007, 0.096 | .09 |  | 0.03 | 0.02 | 0.04 | 0.03 | -0.008, 0.10 | .10 |
| Neuroticism => I Mental Health | 0.12 | 0.02 | 0.28 | 0.03 | 0.21, 0.34 | <.001 |  | 0.12 | 0.02 | 0.27 | 0.03 | 0.20, 0.33 | <.001 |
| SPS => I Mental Health | 0.03 | 0.003 | 0.35 | 0.03 | 0.28, 0.41 | <.001 |  | 0.03 | 0.003 | 0.35 | 0.03 | 0.28, 0.41 | <.001 |
| I CER => I Mental Health | 0.18 | 0.05 | 0.13 | 0.04 | 0.05, 0.20 | .001 |  | 0.22 | 0.05 | 0.16 | 0.04 | 0.08, 0.23 | <.001 |
| I CER × SPS => I Mental Health |  |  |  |  |  |  |  | 0.01 | 0.004 | 0.10 | 0.03 | 0.04, 0.16 | .001 |
| Gender => S Mental Health | 0.02 | 0.05 | 0.02 | 0.05 | -0.08, 0.12 | .67 |  | 0.02 | 0.05 | 0.02 | 0.05 | -0.08, 0.12 | .70 |
| Age => S Mental Health | 0.01 | 0.02 | 0.04 | 0.05 | -0.06, 0.13 | .43 |  | 0.02 | 0.02 | 0.04 | 0.05 | -0.05, 0.13 | .35 |
| Neuroticism => S Mental Health | -0.02 | 0.01 | -0.09 | 0.06 | -0.20, 0.03 | .14 |  | -0.02 | 0.01 | -0.07 | 0.06 | -0.18, 0.04 | .19 |
| SPS => S Mental Health | -0.002 | 0.003 | -0.05 | 0.06 | -0.18, 0.07 | .40 |  | -0.003 | 0.003 | -0.07 | 0.06 | -0.19,0.05 | .23 |
| I CER => S Mental Health | 0.07 | 0.05 | 0.12 | 0.08 | -0.04, 0.28 | .14 |  | 0.06 | 0.05 | 0.10 | 0.08 | -0.06, 0.25 | .22 |
| S CER => S Mental Health | 0.46 | 0.12 | 0.38 | 0.09 | 0.20, 0.55 | <.001 |  | 0.43 | 0.12 | 0.35 | 0.09 | 0.17, 0.52 | <.001 |
| I CER × SPS => S Mental Health |  |  |  |  |  |  |  | -0.008 | 0.004 | -0.14 | 0.07 | -0.28, -0.01 | .03 |
| S CER × SPS => S Mental Health |  |  |  |  |  |  |  | -0.02 | 0.01 | -0.18 | 0.09 | -0.35, -0.003 | .046 |
| *Notes.* SE = standard error, I = intercept, S = slope, SPS = Sensory Processing Sensitivity, CER = Cognitive Emotion Regulation. Gender was used as a dummy variable (1 = men, 2 =women, 3 =unidentified/other). The models in bold type were fitted better to data in this study. | | | | | | | | | | | | | |

Table S7. Parameters estimated in the multivariate latent growth model and the latent moderated equation model with *acceptance* as a predictor

|  | **Without Interaction Model** | | | | | |  | With Interaction Model | | | | | |
| --- | --- | --- | --- | --- | --- | --- | --- | --- | --- | --- | --- | --- | --- |
|  | *b* | *b SE* | β | β *SE* | 95%CI | *p* |  | *b* | *b SE* | β | β *SE* | 95%CI | *p* |
| Gender => I Mental Health | -0.18 | 0.06 | -0.08 | 0.03 | -0.14, -0.03 | .002 |  | -0.17 | 0.06 | -0.08 | 0.03 | -0.14, -0.03 | .002 |
| Age => I Mental Health | 0.03 | 0.02 | 0.04 | 0.03 | -0.01, 0.09 | .16 |  | 0.03 | 0.02 | 0.04 | 0.03 | -0.01, 0.09 | .15 |
| Neuroticism => I Mental Health | 0.13 | 0.02 | 0.27 | 0.03 | 0.21, 0.34 | <.001 |  | 0.13 | 0.01 | 0.27 | 0.03 | 0.21, 0.33 | <.001 |
| SPS => I Mental Health | 0.04 | 0.003 | 0.39 | 0.03 | 0.33, 0.45 | <.001 |  | 0.04 | 0.003 | 0.40 | 0.03 | 0.33, 0.46 | <.001 |
| I CER => I Mental Health | -0.20 | 0.06 | -0.12 | 0.04 | -0.19, -0.05 | .001 |  | -0.19 | 0.07 | -0.12 | 0.04 | -0.19, -0.04 | .003 |
| I CER × SPS => I Mental Health |  |  |  |  |  |  |  | -0.005 | 0.005 | 0.03 | 0.04 | -0.04, 0.10 | .36 |
| Gender => S Mental Health | 0.03 | 0.05 | 0.03 | 0.05 | -0.07, 0.13 | .51 |  | 0.03 | 0.05 | 0.03 | 0.05 | -0.07, 0.13 | .56 |
| Age => S Mental Health | 0.01 | 0.02 | 0.04 | 0.05 | -0.06, 0.13 | .47 |  | 0.01 | 0.02 | 0.04 | 0.05 | -0.06, 0.13 | .47 |
| Neuroticism => S Mental Health | -0.02 | 0.01 | -0.09 | 0.06 | -0.20, 0.03 | .14 |  | -0.02 | 0.01 | -0.08 | 0.06 | -0.19, 0.03 | .16 |
| SPS => S Mental Health | -0.002 | 0.003 | -0.04 | 0.06 | -0.17, 0.08 | .50 |  | -0.002 | 0.003 | -0.06 | 0.06 | -0.17, 0.06 | .36 |
| I CER => S Mental Health | 0.04 | 0.05 | 0.06 | 0.07 | -0.08, 0.19 | .43 |  | 0.04 | 0.05 | 0.05 | 0.07 | -0.09, 0.19 | .47 |
| S CER => S Mental Health | NA | | | | | |  | NA | | | | | |
| I CER × SPS => S Mental Health |  |  |  |  |  |  |  | -0.006 | 0.005 | -0.10 | 0.07 | -0.23, 0.04 | .16 |
| S CER × SPS => S Mental Health | NA | | | | | |  | NA | | | | | |
| *Notes.* SE = standard error, I = intercept, S = slope, SPS = Sensory Processing Sensitivity, CER = Cognitive Emotion Regulation. Gender was used as a dummy variable (1 = men, 2 =women, 3 =unidentified/other). NA = Not available because the slope factor of the strategy was not included in the model. The models in bold type were fitted better to data in this study. | | | | | | | | | | | | | |

Table S8. Parameters estimated in the multivariate latent growth model and the latent moderated equation model with *self-blame* as a predictor

|  | **Without Interaction Model** | | | | | |  | With Interaction Model | | | | | |
| --- | --- | --- | --- | --- | --- | --- | --- | --- | --- | --- | --- | --- | --- |
|  | *b* | *b SE* | β | β *SE* | 95%CI | *p* |  | *b* | *b SE* | β | β *SE* | 95%CI | *p* |
| Gender => I Mental Health | -0.17 | 0.06 | -0.08 | 0.03 | -0.14, -0.03 | .002 |  | -0.17 | 0.06 | -0.08 | 0.03 | -0.14, -0.03 | .002 |
| Age => I Mental Health | 0.04 | 0.02 | 0.05 | 0.03 | -0.005, 0.097 | .08 |  | 0.04 | 0.02 | 0.05 | 0.03 | -0.004, 0.10 | .07 |
| Neuroticism => I Mental Health | 0.13 | 0.02 | 0.28 | 0.03 | 0.22, 0.34 | <.001 |  | 0.12 | 0.02 | 0.27 | 0.03 | 0.21, 0.34 | <.001 |
| SPS => I Mental Health | 0.04 | 0.003 | 0.37 | 0.03 | 0.30, 0.43 | <.001 |  | 0.04 | 0.003 | 0.37 | 0.03 | 0.30, 0.43 | <.001 |
| I CER => I Mental Health | 0.11 | 0.05 | 0.08 | 0.04 | 0.01, 0.15 | .03 |  | 0.12 | 0.05 | 0.09 | 0.04 | 0.02, 0.16 | .02 |
| I CER × SPS => I Mental Health |  |  |  |  |  |  |  | 0.006 | 0.004 | 0.05 | 0.03 | -0.01, 0.11 | .13 |
| Gender => S Mental Health | 0.02 | 0.05 | 0.02 | 0.05 | -0.08, 0.12 | .70 |  | 0.02 | 0.05 | 0.02 | 0.05 | -0.08, 0.12 | .73 |
| Age => S Mental Health | 0.01 | 0.02 | 0.03 | 0.05 | -0.06, 0.13 | .50 |  | 0.01 | 0.02 | 0.03 | 0.05 | -0.06, 0.13 | .48 |
| Neuroticism => S Mental Health | -0.02 | 0.01 | -0.09 | 0.06 | -0.21, 0.02 | .12 |  | -0.01 | 0.01 | -0.07 | 0.06 | -0.18, 0.04 | .21 |
| SPS => S Mental Health | -0.002 | 0.003 | -0.05 | 0.06 | -0.18, 0.07 | .39 |  | -0.003 | 0.003 | -0.06 | 0.06 | -0.18, 0.07 | .36 |
| I CER => S Mental Health | 0.16 | 0.13 | 0.26 | 0.21 | -0.15, 0.67 | .22 |  | 0.08 | 0.06 | 0.14 | 0.09 | -0.04, 0.32 | .13 |
| S CER => S Mental Health | 1.67 | 1.71 | 0.58 | 0.33 | -0.08, 1.23 | .08 |  | 0.76 | 0.50 | 0.32 | 0.20 | -0.06, 0.71 | .10 |
| I CER × SPS => S Mental Health |  |  |  |  |  |  |  | -0.007 | 0.004 | -0.13 | 0.07 | -0.27, 0.02 | .09 |
| S CER × SPS => S Mental Health |  |  |  |  |  |  |  | -0.05 | 0.03 | -0.22 | 0.14 | -0.49, 0.06 | .12 |
| *Notes.* SE = standard error, I = intercept, S = slope, SPS = Sensory Processing Sensitivity, CER = Cognitive Emotion Regulation. Gender was used as a dummy variable (1 = men, 2 =women, 3 =unidentified/other). The models in bold type were fitted better to data in this study. | | | | | | | | | | | | | |

Table S9. Parameters estimated in the multivariate latent growth model and the latent moderated equation model with *positive refocusing* as a predictor

|  | **Without Interaction Model** | | | | | |  | With Interaction Model | | | | | |
| --- | --- | --- | --- | --- | --- | --- | --- | --- | --- | --- | --- | --- | --- |
|  | *b* | *b SE* | β | β *SE* | 95%CI | *p* |  | *b* | *b SE* | β | β *SE* | 95%CI | *p* |
| Gender => I Mental Health | -0.17 | 0.06 | -0.08 | 0.03 | -0.14, -0.03 | .002 |  | -0.17 | 0.06 | -0.08 | 0.03 | -0.13, -0.03 | .003 |
| Age => I Mental Health | 0.03 | 0.02 | 0.04 | 0.03 | -0.01, 0.09 | .14 |  | 0.03 | 0.02 | 0.03 | 0.03 | -0.01, 0.09 | .14 |
| Neuroticism => I Mental Health | 0.12 | 0.02 | 0.27 | 0.03 | 0.20, 0.33 | <.001 |  | 0.12 | 0.02 | 0.27 | 0.03 | 0.20, 0.33 | <.001 |
| SPS => I Mental Health | 0.04 | 0.003 | 0.38 | 0.03 | 0.32, 0.45 | <.001 |  | 0.04 | 0.003 | 0.39 | 0.03 | 0.32, 0.45 | <.001 |
| I CER => I Mental Health | -0.16 | 0.05 | -0.12 | 0.04 | -0.19, -0.05 | .001 |  | -0.16 | 0.05 | -0.12 | 0.04 | -0.19, -0.05 | .001 |
| I CER × SPS => I Mental Health |  |  |  |  |  |  |  | 0.003 | 0.004 | 0.03 | 0.03 | -0.04, 0.09 | .44 |
| Gender => S Mental Health | 0.04 | 0.05 | 0.04 | 0.05 | -0.06, 0.14 | .45 |  | 0.03 | 0.05 | 0.03 | 0.05 | -0.07, 0.14 | .50 |
| Age => S Mental Health | 0.01 | 0.02 | 0.03 | 0.05 | -0.06, 0.13 | .51 |  | 0.01 | 0.02 | 0.04 | 0.05 | -0.06, 0.13 | .45 |
| Neuroticism => S Mental Health | -0.02 | 0.01 | -0.11 | 0.06 | -0.22, 0.01 | .08 |  | -0.02 | 0.01 | -0.10 | 0.06 | -0.22, 0.01 | .08 |
| SPS => S Mental Health | -0.001 | 0.003 | -0.03 | 0.07 | -0.16, 0.09 | .61 |  | -0.003 | 0.003 | -0.06 | 0.06 | -0.18, 0.06 | .34 |
| I CER => S Mental Health | -0.03 | 0.05 | -0.06 | 0.08 | -0.22, 0.10 | .49 |  | -0.03 | 0.05 | -0.05 | 0.08 | -0.21, 0.12 | .59 |
| S CER => S Mental Health | -0.09 | 0.11 | -0.08 | 0.10 | -0.28, 0.12 | .45 |  | -0.10 | 0.12 | -0.09 | 0.10 | -0.29, 0.12 | .41 |
| I CER × SPS => S Mental Health |  |  |  |  |  |  |  | -0.009 | 0.004 | -0.17 | 0.07 | -0.31, -0.03 | .02 |
| S CER × SPS => S Mental Health |  |  |  |  |  |  |  | -0.01 | 0.009 | -0.10 | 0.08 | -0.26, 0.07 | .26 |
| *Notes.* SE = standard error, I = intercept, S = slope, SPS = Sensory Processing Sensitivity, CER = Cognitive Emotion Regulation. Gender was used as a dummy variable (1 = men, 2 =women, 3 =unidentified/other). The models in bold type were fitted better to data in this study. | | | | | | | | | | | | | |

Table S10. Parameters estimated in the multivariate latent growth model and the latent moderated equation model with *blaming others* as a predictor

|  | Without Interaction Model | | | | | |  | **With Interaction Model** | | | | | |
| --- | --- | --- | --- | --- | --- | --- | --- | --- | --- | --- | --- | --- | --- |
|  | *b* | *b SE* | β | β *SE* | 95%CI | *p* |  | *b* | *b SE* | β | β *SE* | 95%CI | *p* |
| Gender => I Mental Health | -0.18 | 0.06 | -0.08 | 0.03 | -0.14, -0.03 | .002 |  | -0.17 | 0.06 | -0.08 | 0.03 | -0.14, -0.03 | .002 |
| Age => I Mental Health | 0.03 | 0.02 | 0.04 | 0.03 | -0.01, 0.09 | .11 |  | 0.03 | 0.02 | 0.04 | 0.03 | -0.01, 0.09 | .11 |
| Neuroticism => I Mental Health | 0.13 | 0.02 | 0.28 | 0.03 | 0.22, 0.35 | <.001 |  | 0.13 | 0.02 | 0.29 | 0.03 | 0.22, 0.35 | <.001 |
| SPS => I Mental Health | 0.04 | 0.003 | 0.38 | 0.03 | 0.31, 0.44 | <.001 |  | 0.04 | 0.003 | 0.38 | 0.03 | 0.31, 0.44 | <.001 |
| I CER => I Mental Health | 0.05 | 0.05 | 0.03 | 0.04 | -0.04, 0.10 | .37 |  | 0.05 | 0.05 | 0.03 | 0.04 | -0.04, 0.10 | .34 |
| I CER × SPS => I Mental Health |  |  |  |  |  |  |  | 0.003 | 0.004 | 0.03 | 0.03 | -0.04, 0.09 | .43 |
| Gender => S Mental Health | 0.03 | 0.05 | 0.03 | 0.05 | -0.07, 0.13 | .52 |  | 0.02 | 0.05 | 0.03 | 0.05 | -0.07, 0.12 | .60 |
| Age => S Mental Health | 0.01 | 0.02 | 0.04 | 0.05 | -0.06, 0.13 | .44 |  | 0.02 | 0.02 | 0.04 | 0.05 | -0.05, 0.14 | .38 |
| Neuroticism => S Mental Health | -0.02 | 0.01 | -0.08 | 0.06 | -0.20, 0.04 | .18 |  | -0.02 | 0.01 | -0.08 | 0.06 | -0.19, 0.03 | .16 |
| SPS => S Mental Health | -0.001 | 0.003 | -0.03 | 0.06 | -0.16, 0.09 | .60 |  | -0.004 | 0.003 | -0.09 | 0.06 | -0.22, 0.04 | .16 |
| I CER => S Mental Health | 0.02 | 0.06 | 0.03 | 0.09 | -0.15, 0.20 | .78 |  | 0.009 | 0.06 | 0.01 | 0.08 | -0.15, 0.17 | .87 |
| S CER => S Mental Health | 0.44 | 0.30 | 0.26 | 0.15 | -0.04, 0.55 | .10 |  | 0.37 | 0.22 | 0.22 | 0.12 | -0.009, 0.45 | .06 |
| I CER × SPS => S Mental Health |  |  |  |  |  |  |  | -0.01 | 0.005 | -0.21 | 0.08 | -0.36, -0.05 | .008 |
| S CER × SPS => S Mental Health |  |  |  |  |  |  |  | -0.06 | 0.02 | -0.36 | 0.09 | -0.53, -0.19 | <.001 |
| *Notes.* SE = standard error, I = intercept, S = slope, SPS = Sensory Processing Sensitivity, CER = Cognitive Emotion Regulation. Gender was used as a dummy variable (1 = men, 2 =women, 3 =unidentified/other). The models in bold type were fitted better to data in this study. | | | | | | | | | | | | | |

Table S11. Parameters estimated in the multivariate latent growth model and the latent moderated equation model with *catastrophizing* as a predictor

|  | Without Interaction Model | | | | | |  | **With Interaction Model** | | | | | |
| --- | --- | --- | --- | --- | --- | --- | --- | --- | --- | --- | --- | --- | --- |
|  | *b* | *b SE* | β | β *SE* | 95%CI | *p* |  | *b* | *b SE* | β | β *SE* | 95%CI | *p* |
| Gender => I Mental Health | -0.15 | 0.05 | -0.08 | 0.03 | -0.13, -0.02 | .005 |  | -0.15 | 0.05 | -0.08 | 0.03 | -0.13, -0.02 | .005 |
| Age => I Mental Health | 0.03 | 0.02 | 0.04 | 0.03 | -0.01, 0.09 | .15 |  | 0.03 | 0.02 | 0.04 | 0.03 | -0.01, 0.09 | .16 |
| Neuroticism => I Mental Health | 0.12 | 0.02 | 0.27 | 0.03 | 0.20, 0.33 | <.001 |  | 0.12 | 0.02 | 0.26 | 0.03 | 0.20, 0.33 | <.001 |
| SPS => I Mental Health | 0.03 | 0.003 | 0.34 | 0.04 | 0.27, 0.40 | <.001 |  | 0.03 | 0.003 | 0.34 | 0.03 | 0.27, 0.41 | <.001 |
| I CER => I Mental Health | 0.25 | 0.05 | 0.21 | 0.04 | 0.14, 0.29 | <.001 |  | 0.26 | 0.05 | 0.22 | 0.04 | 0.14, 0.29 | <.001 |
| I CER × SPS => I Mental Health |  |  |  |  |  |  |  | 0.006 | 0.003 | 0.06 | 0.03 | -0.001, 0.12 | .06 |
| Gender => S Mental Health | 0.02 | 0.05 | 0.02 | 0.05 | -0.07, 0.12 | .63 |  | 0.01 | 0.05 | 0.01 | 0.05 | -0.09, 0.11 | .80 |
| Age => S Mental Health | 0.01 | 0.02 | 0.04 | 0.05 | -0.06, 0.13 | .45 |  | 0.01 | 0.02 | 0.04 | 0.05 | -0.05, 0.14 | .37 |
| Neuroticism => S Mental Health | -0.02 | 0.01 | -0.09 | 0.06 | -0.21, 0.03 | .13 |  | -0.02 | 0.01 | -0.08 | 0.06 | -0.19, 0.04 | .18 |
| SPS => S Mental Health | -0.002 | 0.003 | -0.05 | 0.06 | -0.17, 0.08 | .47 |  | -0.004 | 0.003 | -0.09 | 0.06 | -0.21, 0.04 | .17 |
| I CER => S Mental Health | 0.07 | 0.04 | 0.13 | 0.07 | -0.02, 0.27 | .08 |  | 0.07 | 0.04 | 0.13 | 0.07 | -0.01, 0.28 | .07 |
| S CER => S Mental Health | 0.18 | 0.05 | 0.31 | 0.09 | 0.14, 0.48 | <.001 |  | 0.31 | 0.11 | 0.26 | 0.09 | 0.08, 0.44 | .005 |
| I CER × SPS => S Mental Health |  |  |  |  |  |  |  | -0.006 | 0.003 | -0.13 | 0.07 | -0.26, 0.004 | .06 |
| S CER × SPS => S Mental Health |  |  |  |  |  |  |  | -0.02 | 0.009 | -0.21 | 0.08 | -0.37, -0.05 | .009 |
| *Notes.* SE = standard error, I = intercept, S = slope, SPS = Sensory Processing Sensitivity, CER = Cognitive Emotion Regulation. Gender was used as a dummy variable (1 = men, 2 =women, 3 =unidentified/other). The models in bold type were fitted better to data in this study. | | | | | | | | | | | | | |

Table S12. Parameters estimated in the multivariate latent growth model and the latent moderated equation model with *refocusing on planning* as a predictor

|  | Without Interaction Model | | | | | |  | **With Interaction Model** | | | | | |
| --- | --- | --- | --- | --- | --- | --- | --- | --- | --- | --- | --- | --- | --- |
|  | *b* | *b SE* | β | β *SE* | 95%CI | *p* |  | *b* | *b SE* | β | β *SE* | 95%CI | *p* |
| Gender => I Mental Health | -0.19 | 0.06 | -0.09 | 0.03 | -0.14, -0.04 | .001 |  | -0.19 | 0.06 | -0.09 | 0.03 | -0.14, -0.04 | .001 |
| Age => I Mental Health | 0.03 | 0.02 | 0.04 | 0.03 | -0.01, 0.09 | .13 |  | 0.03 | 0.02 | 0.04 | 0.03 | -0.01, 0.09 | .13 |
| Neuroticism => I Mental Health | 0.12 | 0.02 | 0.25 | 0.03 | 0.19, 0.32 | <.001 |  | 0.12 | 0.02 | 0.25 | 0.03 | 0.19, 0.32 | <.001 |
| SPS => I Mental Health | 0.04 | 0.003 | 0.41 | 0.03 | 0.35, 0.47 | <.001 |  | 0.04 | 0.003 | 0.41 | 0.03 | 0.35, 0.47 | <.001 |
| I CER => I Mental Health | -0.21 | 0.05 | -0.16 | 0.03 | -0.22, -0.09 | <.001 |  | -0.21 | 0.05 | -0.15 | 0.04 | -0.22, -0.08 | <.001 |
| I CER × SPS => I Mental Health |  |  |  |  |  |  |  | 0.000 | 0.004 | 0.001 | 0.03 | -0.06, 0.06 | .98 |
| Gender => S Mental Health | 0.04 | 0.05 | 0.04 | 0.05 | -0.06, 0.14 | .44 |  | 0.04 | 0.05 | 0.04 | 0.05 | -0.06, 0.14 | .42 |
| Age => S Mental Health | 0.01 | 0.02 | 0.03 | 0.05 | -0.06, 0.13 | .51 |  | 0.01 | 0.02 | 0.04 | 0.05 | -0.06, 0.13 | .44 |
| Neuroticism => S Mental Health | -0.02 | 0.01 | -0.08 | 0.06 | -0.20, 0.04 | .18 |  | -0.01 | 0.01 | -0.07 | 0.06 | -0.18, 0.04 | .20 |
| SPS => S Mental Health | -0.002 | 0.003 | -0.05 | 0.07 | -0.18, 0.08 | .42 |  | -0.003 | 0.003 | -0.07 | 0.06 | -0.19, 0.05 | .26 |
| I CER => S Mental Health | 0.04 | 0.04 | 0.07 | 0.07 | -0.07, 0.20 | .33 |  | 0.03 | 0.04 | 0.05 | 0.07 | -0.08, 0.18 | .42 |
| S CER => S Mental Health | -0.15 | 0.12 | -0.11 | 0.09 | -0.28, 0.07 | .23 |  | -0.14 | 0.12 | -0.10 | 0.08 | -0.27, 0.06 | .22 |
| I CER × SPS => S Mental Health |  |  |  |  |  |  |  | -0.006 | 0.004 | -0.11 | 0.07 | -0.24, 0.02 | .11 |
| S CER × SPS => S Mental Health |  |  |  |  |  |  |  | -0.02 | 0.008 | -0.16 | 0.07 | -0.29, -0.03 | .02 |
| *Notes.* SE = standard error, I = intercept, S = slope, SPS = Sensory Processing Sensitivity, CER = Cognitive Emotion Regulation. Gender was used as a dummy variable (1 = men, 2 =women, 3 =unidentified/other). The models in bold type were fitted better to data in this study. | | | | | | | | | | | | | |
